# Bond Strength of a Self-Adhesive Universal Resin Cement to Enamel and Dentin with or without an Adhesive Bonding Agent

**DOI:** 10.4317/jced.63087

**Published:** 2025-09-01

**Authors:** Jeremiah Gossett, Stephen C Arnason, Andrew W Ellis, Kraig S Vandewalle

**Affiliations:** 1 DMD, MS. Maj, USAF, DC. Dental Clinic, 56th Medical Group. 7219 North Litchfield Road, Building #1130. Luke Air Force Base, AZ, USA 85309; 2 DDS, MS.Lt Col, USAF, DC. 59th Dental Support Squadron Commander. 2133 Pepperrell St, Joint Base San Antonio - Lackland, TX, USA 78236. Uniformed Services University of the Health Sciences, Bethesda, MD, USA, 20814; 3 DMD, MS. Lt Col, USAF, DC. Program Director, Advanced Education in General Dentistry Residency. 151 Bodin Circle, Travis AFB, CA, USA 94535. Uniformed Services University of the Health Sciences, Bethesda, MD, USA, 20814; 4 DDS, MS. Col (ret), USAF, DC. Director, Dental Research, Advanced Education in General Dentistry Residency. AF Postgraduate Dental School. 1615 Truemper St, Joint Base San Antonio - Lackland, TX, USA 78236. Uniformed Services University of the Health Sciences, Bethesda, MD, USA, 20814

## Abstract

**Background:**

The recent advent of universal resin cements provides clinicians with a versatile material that can function either as a self-adhesive or as an adhesive resin cement, depending on the specific clinical requirements. This investigation aimed to assess the shear bond strength of a contemporary self-adhesive universal resin cement (Panavia SA Cement Universal, Kuraray) when applied to enamel or dentin. For comparative analysis, its performance was benchmarked against an established control adhesive resin cement (NX3 Nexus, Kerr), both with and without the supplementary application of an adhesive bonding agent (Clearfil Universal Bond Quick, Kuraray).

**Material and Methods:**

Human molars were mounted using PVC pipes. Coronal tooth structure was removed to expose enamel or dentin. A bonding agent was applied to half of the specimens per cement and light cured. The specimens were placed in a jig and secured beneath a 2.4mm diameter mold. The resin cements were mixed according to the manufacturer’s instructions, applied into the mold, and light cured (*n*=12). Prior to shear bond strength testing and failure mode analysis, all specimens underwent thermocycling. The resulting shear bond strength data for both enamel or dentin were statistically analyzed using one-way ANOVA and Tukey’s post hoc tests (α=0.05).

**Results:**

Our findings indicated that the universal resin cement’s bond strength to enamel was not significantly influenced by the presence or absence of a bonding agent. Furthermore, its performance on enamel was comparable to that of the adhesive resin cement when a bonding agent was utilized. We observed a similar distribution of cohesive and mixed failures for the universal resin cement, regardless of whether a bonding agent was used, and for the adhesive resin cement combined with a bonding agent. Conversely, for dentin, the universal resin cement demonstrated significantly greater bond strength when a bonding agent was incorporated. However, even with a bonding agent, both universal resin cement applications (with and without bonding agent) yielded significantly lower bond strengths compared to the adhesive resin cement used with a bonding agent. Notably, a higher incidence of adhesive failures was recorded for both the adhesive and universal resin cements on dentin when no bonding agent was employed.

**Conclusions:**

While the versatility of the novel universal resin cement (Panavia SA Cement Universal) allows for both adhesive and self-adhesive applications, our findings indicate that the adhesive resin cement (NX3 Nexus), when used with a bonding agent, consistently delivered the highest shear bond strength to dentin. However, its bond strength to enamel was comparable across the tested materials.

** Key words:**Universal Resin Cement, Bonding Agent, Enamel, Dentin.

## Introduction

The efficacy of resin cement is paramount for the longevity and success of indirect dental restorations in modern dentistry [1,2]. Historically, these cements have been categorized as either adhesive or self-adhesive [3,4]. Adhesive resin cements require a distinct bonding step, which can add to both technique sensitivity and chair time. Nevertheless, they remain the preferred choice in clinical scenarios where tooth preparations offer limited retention and resistance [4]. Conversely, self-adhesive resin cements streamline the procedure by functioning as a single-step system, eliminating the need for separate etchants, primers, or bonding agents, making them ideal for preparations with inherent retention [5]. These self-etching cements initiate bonding through a low pH environment, enabling them to etch dentin and form both micromechanical and chemical bonds with hydroxyapatite during polymerization, often via monomers like 10-methacryloyloxydecyl dihydrogen phosphate (10-MDP). Despite the procedural convenience offered by self-adhesive options, adhesive resin cements have consistently shown superior adhesive performance, a finding supported by a recent systematic review of laboratory studies [6]. This enhanced performance is likely due to the deeper penetration achieved by primers and bonding agents into the demineralized dentin substrate [6].

The dental landscape has recently welcomed universal resin cements, a versatile class of materials that can be employed as either a self-adhesive or an adhesive resin cement, based on the individual clinical presentation. Illustrating this trend, Kuraray (Tokyo, Japan) developed Panavia SA Cement Universal. This particular dual-cure, self-adhesive, universal resin cement provides the option of use without a bonding agent (self-adhesive mode) or with a bonding agent (adhesive resin cement mode) [7]. Its formulation includes a hydrophilic paste incorporating 10-MDP, a component that significantly contributes to its robust bond strength with tooth structure [7].

While universal resin cements are gaining popularity in clinical practice, comprehensive data on their bond strength to tooth structure, particularly when a separate bonding agent is or isn’t used, remains limited [8,9]. Furthermore, Panavia SA Cement Universal specifically lacks evaluation under these parameters. Consequently, the primary objective of this investigation is to determine the shear bond strength of this new dual-cure, self-adhesive universal resin cement on enamel and dentin. This will involve a direct comparison with a conventional dual-cure adhesive resin cement, NX3 Nexus (Kerr, Orange, CA, USA) [10]. assessing its application both with and without a universal self-etching bonding agent (Clearfil Universal Bond Quick, Kuraray). The study’s null hypotheses propose no significant differences in bond strength to either (1) enamel or (2) dentin, irrespective of the resin cement type or the use of a bonding agent.

## Material and Methods

This study was conducted using 96 extracted human third molars that exhibited no signs of caries. After initial cleaning, each tooth was then secured within polyvinyl chloride pipes using dental acrylic, with the crown positioned to remain accessible. For the creation of dentin specimens, 48 teeth were precisely cut using a diamond saw (Isomet, Buehler, Lake Forest, IL, USA). At least 2 mm of the coronal tooth material was removed to ensure optimal exposure of the dentin bonding surface. To confirm the complete absence of enamel, each specimen was carefully examined under a stereomicroscope. Finally, a consistent smear layer was achieved on the dentin through 20 passes each of 120- and 600-grit silicon carbide paper.

To obtain enamel specimens, a distinct preparation method was employed on 48 teeth using the diamond saw. In contrast to dentin preparation, less than 2 mm of coronal tooth structure was removed to specifically expose a fresh enamel surface for bonding. Prior to further steps, each specimen underwent stereomicroscopic examination to guarantee the absence of any dentinal layer at the designated bonding areas. Subsequently, a smear layer was generated on the enamel surface by applying the silicon carbide paper for 20 passes each, mirroring the previous protocol.

The 48 dentin and 48 enamel tooth specimens were systematically allocated into two subgroups of 24 each. For each surface type (dentin and enamel), 24 specimens were assigned to Panavia SA Cement Universal, and the remaining 24 specimens were designated for NX3 Nexus resin cement. Within each cement group, half of the specimens (*n*=12) were tested in conjunction with an adhesive bonding agent, while the other 12 specimens were evaluated with the cement applied directly, without a bonding agent. A comprehensive list of the resin cement and bonding agent components used in this investigation is provided in  Table 1.

For the adhesive bonding groups, Clearfil Universal Bond Quick was applied per the manufacturer’s instructions to the dentin or enamel surface. The bonding agent was applied to the dentin specimens with a rubbing motion, air-dried for 5 seconds, and then light cured (Valo Grand, Ultradent Products, South Jordan, UT, USA) for 10 seconds in standard mode. For the enamel specimens, the surface was etched with phosphoric acid (K-Etchant, Kuraray) for 10 seconds - to simulate a selective etch mode – and then rinsed and dried before the placement of the universal bonding agent. A jig containing a 2.4mm diameter mold was placed onto the bonded dentin or enamel surfaces and secured into place. Each of the two resin cements were separately mixed according to the manufacturer’s instructions and placed into the mold. The specimens were light cured for 60 seconds in standard power mode.

For the self-adhesive groups, each of the two resin cements were utilized without an adhesive bonding agent. The jig was placed onto the dentin or enamel surface and secured into place. The two chosen resin cements were prepared in accordance with their respective manufacturers’ guidelines and then applied to the molds as previously described. Each specimen underwent light-curing for a total of 60 seconds. Following cement application, specimens were stored in distilled water at 37°C and after a 24-hour initial curing period, samples were subjected to 2500 thermocycles in distilled water, alternating between 5.5°C and 55°C, with a 30-second dwell time at each temperature (NesLab Ex10, Portsmouth, NH, USA). Subsequently, a knife-edge probe applied perpendicular load at the tooth-cement interface using a universal testing machine (Instron, Model 5439, Norwood, MA, USA) at a crosshead speed of 1.0 mm/minute until failure. Shear bond strength values, expressed in megapascals (MPa), were derived by dividing the peak failure load (Newtons) by the specimen’s surface area. The mean and standard deviation were computed for each group. Data analysis involved a one-way analysis of variance (ANOVA) and Tukey’s post hoc tests to assess the influence of cement type and adhesive mode on the resin cement’s bond strength to enamel or dentin (α=0.05). Post-testing, each specimen was examined under a stereomicroscope to classify the failure mode as either adhesive (at the resin cement-tooth interface), cohesive (within the resin cement), or mixed (combining adhesive and cohesive elements). All statistical computations were performed using statistical software (SAS, Cary, NC, USA).

## Results

The results of the one-way ANOVA and Tukey’s post hoc tests found that there were no significant differences (*p*>0.930) in bond strength to enamel between the universal resin cement with (17.07 ± 4.67 MPa) or without (16.79 ± 4.51 MPa) the use of a bonding agent and the adhesive resin cement with the use of a bonding agent (17.89 ± 5.04 MPa). The adhesive resin cement without a bonding agent (7.05 ± 3.45 MPa) had the lowest bond strength to enamel and was significantly lower (*p*<0.001) than all the other resin cement combinations to enamel. To dentin, the universal resin cement had significantly greater bond strength (*p*=0.016) with the use of a bonding agent (9.82 ± 3.04 MPa) than without (6.55 ± 2.49 MPa), but both were significantly less (*p*=0.02 and *p*<0.001, respectively) than the adhesive resin cement with a bonding agent (13.01 ± 3.28 MPa). The bond strength of the adhesive resin cement to dentin without a bonding agent (0.90 ± 0.50 MPa) was significantly less (*p*<0.001) than all the other groups ( Table 2).

**Figure 1:**
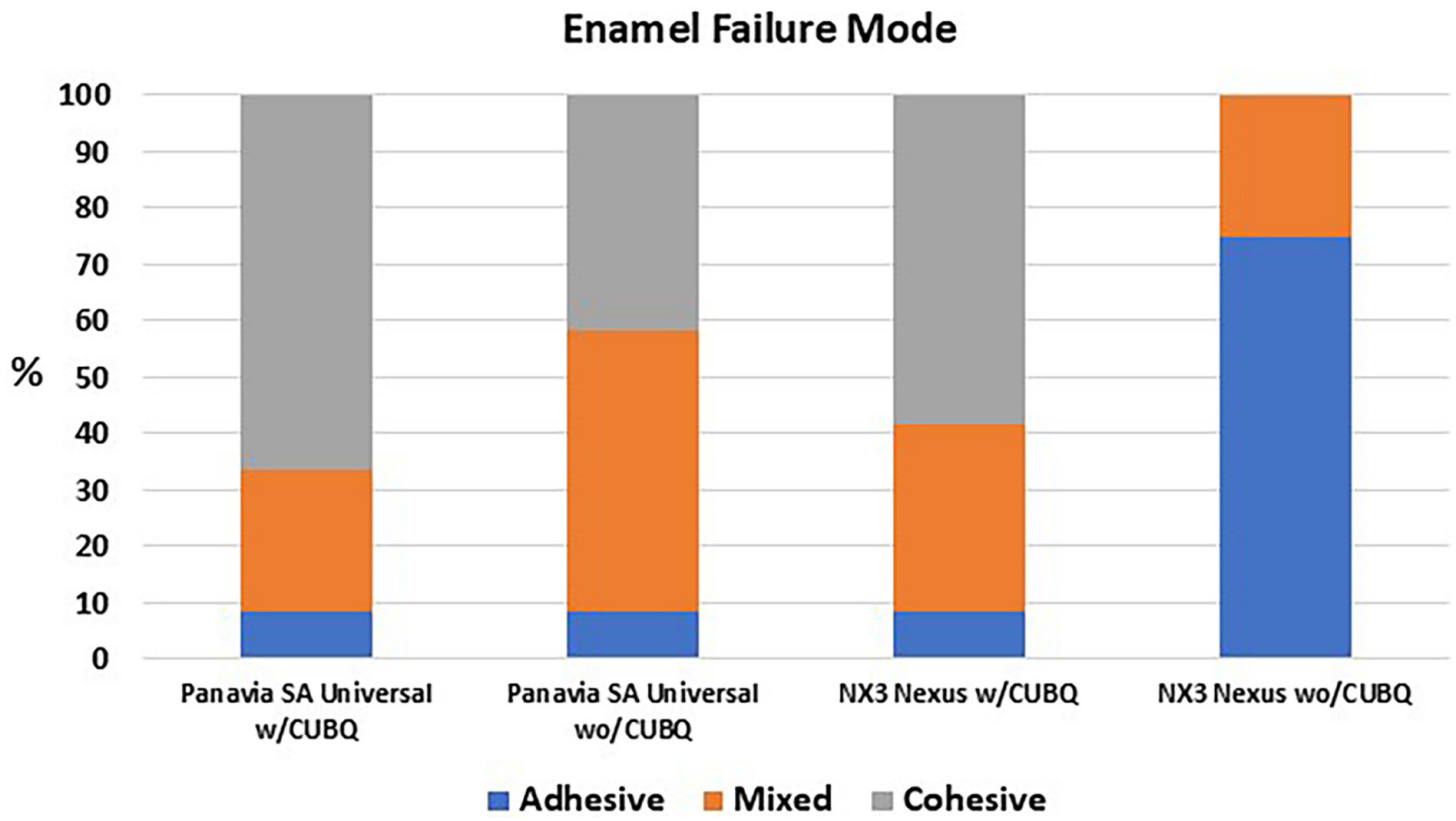
Failure mode of the resin cements to enamel.

**Figure 2:**
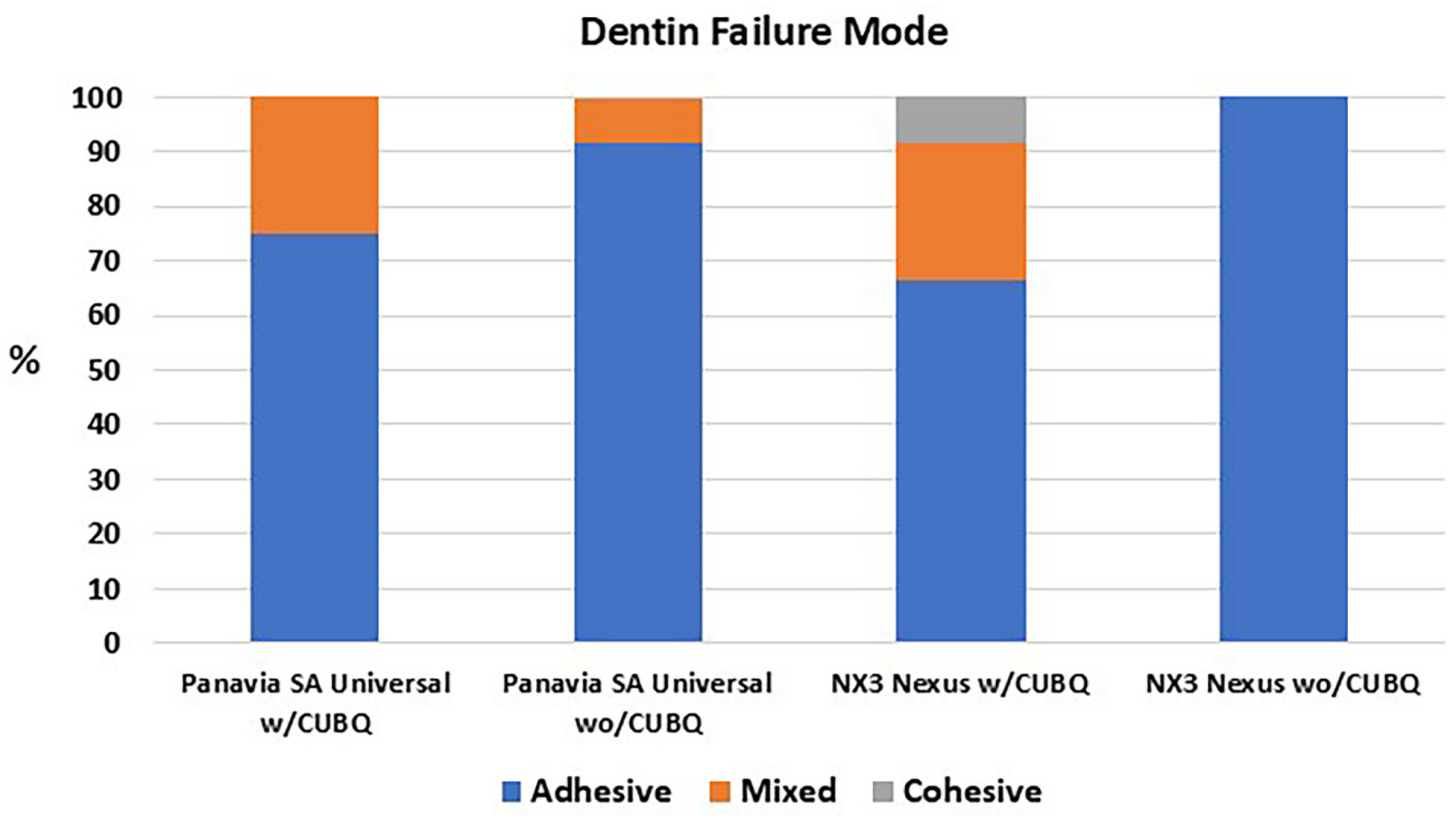
Failure mode of the resin cements to dentin.

To enamel, a similar percentage of cohesive and mixed failures were seen with the adhesive resin cement with a bonding agent (91.6%) and the universal resin cement with (91.6%) and without (91.6%) a bonding agent, (Fig. 1). The adhesive resin cement without a bonding agent had primarily adhesive failures (75%). To dentin, more adhesive failures were seen without the use of a bonding agent for both the adhesive resin cement (100%) and the universal resin cement (91.6%), (Fig. 2).

## Discussion

Our study led to the rejection of the first null hypothesis, as significant differences in bond strength to enamel were observed. Notably, the universal resin cement’s bond strength to enamel did not vary significantly whether a bonding agent was included or not. Furthermore, its performance was statistically comparable to that of the adhesive resin cement when a bonding agent was applied. This suggests that maximum bond strength to enamel may have been achieved across these three groups. This interpretation is supported by the failure mode analysis, which revealed that 91.6% of failures were mixed or cohesive, types typically associated with robust adhesion compared to purely adhesive failures [11]. As anticipated, the adhesive resin cement exhibited significantly lower bond strength to enamel when used without a bonding agent. It’s important to note that NX3 Nexus is not intended for use without a bonding agent but served as a negative control in this investigation [10].

The second null hypothesis was similarly rejected, due to observed differences in bond strength to dentin. The universal resin cement demonstrated a noTable 50% increase in bond strength to dentin when applied with a bonding agent compared to its self-adhesive mode. Nevertheless, in both scenarios, its bond strength was significantly less than that achieved by the adhesive resin cement used with a bonding agent. The increased incidence of adhesive failures in the absence of a bonding agent when bonding to dentin further underscores the critical role of these agents in enhancing adhesive performance. The improved performance of both resin cements when a bonding agent was present can likely be ascribed to the bonding agent’s capacity for deeper penetration into the demineralized dentin substrate, a mechanism well-documented in existing literature [6]. In comparison to our findings, the shear bond strength of RelyX Universal (3M, St. Paul, MN, USA), another universal resin cement, to dentin presented somewhat mixed results in prior studies regarding the use of a bonding agent. Atalay *et al*. observed only a modest 8% increase in dentin shear bond strength when this universal resin cement was combined with a universal bonding agent (Scotchbond Universal, 3M) [8]. Conversely, Andrews *et al*. reported a substantial 141% increase in bond strength to dentin with the identical 3M material combination [9].

This marks the first investigation to evaluate the newer self-adhesive universal resin cement, Panavia SA Cement Universal, under conditions both with and without an adhesive bonding agent. The observed differences in bond strength between Panavia SA Cement Universal and NX3 Nexus are likely attribuTable to their distinct chemical compositions and adhesion mechanisms. Panavia SA Cement Universal’s formulation primarily includes 10-MDP, in addition to Bis-GMA and TEGDMA [7]. The 10-MDP monomer is well-known for its capacity to form a robust chemical bond through interaction with hydroxyapatite on both enamel and dentin. However, due to the inherent absence of primer components found in separate adhesive systems, self-adhesive cements generally have a limited ability to deeply infiltrate demineralized dentin and establish a strong hybrid layer without the aid of an additional bonding agent [6]. In contrast, NX3 Nexus relies on traditional dimethacrylate chemistry, lacking 10-MDP [10]. When used with its required bonding agent, NX3 Nexus benefits from the enhanced micromechanical and chemical interactions facilitated by Clearfil Universal Bond Quick—a 10-MDP containing bonding agent—resulting in superior bond strength to dentin [7]. Without a bonding agent, NX3 Nexus’s adhesive capability is compromised, leading to a significant reduction in adhesion, particularly to dentin.

The universal resin cement, Panavia SA Cement Universal, demonstrated comparable bond strength to enamel as the adhesive resin cement, NX3 Nexus, when the latter was used with a bonding agent. Furthermore, Panavia SA Cement Universal exhibited significantly greater bond strength to dentin than the adhesive resin cement used without a bonding agent. These findings suggest that Panavia SA Cement Universal offers flexibility for use in either a self-adhesive or adhesive mode, depending on the specific clinical presentation.

For certain clinical indications, such as non-retentive partial-coverage ceramic restorations like inlays and onlays, the adhesive mode may remain the preferred choice [12,13]. Conversely, for more retentive all-ceramic crown restorations, the self-adhesive mode with the universal resin cement could provide adequate adhesion, offering advantages in terms of ease of use, placement, and cleanup [14,15]. However, considering the limitations of this study, achieving maximum bond strength to dentin may still necessitate the clinical preference for the conventional adhesive resin cement, NX3 Nexus, with a bonding agent.

A significant limitation of this *in vitro* study is its evaluation of only two resin cement types and a single bonding agent. The generalizability of these findings is therefore limited, as the performance of other universal resin cements or alternative bonding agents may vary significantly due to differences in their chemical compositions, pH, and interaction with tooth substrates.

Furthermore, several methodological assumptions, while standard in laboratory settings, introduce limitations when extrapolating to the complex oral environment. The application of the bonding agent and resin cements occurred under controlled laboratory conditions (e.g., controlled humidity, temperature, precise curing light parameters). This ideal environment minimizes confounding variables but does not fully account for the challenges of clinical practice, such as saliva contamination, moisture control issues, or variations in operator technique, which can all negatively impact bond strength [11]. While thermocycling simulates temperature fluctuations in the oral cavity, the chosen number of cycles (2500) and dwell times, while common, may not fully replicate long-term oral degradation and fatigue phenomena [11]. Additionally, shear bond strength testing is a widely accepted method for evaluating adhesive performance. However, it applies a unidirectional force, which may not fully represent the complex multi-directional stresses (e.g., tensile, compressive, and shear components) experienced by restorations *in vivo* [12]. Finally, the absence of physiological pulp pressure, dynamic occlusal loads, continuous exposure to oral fluids, and microbial challenges in a controlled laboratory setting means that the reported bond strengths, while valuable for comparative purposes, may not directly translate to the actual clinical longevity and performance of restorations [15]. Future investigations are essential to explore the clinical performance of indirect restorations bonded to tooth structure using the newer universal resin cements, both with and without a bonding agent.

## Conclusions

While the novel Panavia SA Cement Universal offers the flexibility of both adhesive and self-adhesive applications, the NX3 Nexus adhesive resin cement, when paired with a bonding agent, consistently achieved the strongest shear bond strength to dentin. Its performance on enamel, however, was found to be comparable to the other materials tested. While the current study provides valuable initial insights, a systematic and multi-faceted research approach is needed to fully understand the clinical performance, optimal usage, and long-term durability of the expanding range of universal resin cements and their interactions with various bonding agents.

## Figures and Tables

**Table 1:** Table Components of materials used in this study.

Material Name	Manufacturer	Components
NX3	Kerr	Base: barium aluminoborosilicate glass, ytterbium fluoride, ethoxylated bisphenol-A dimethacrylate, urethane dimethacrylate, triethylene glycol dimethacrylate, hydroxymethyl methacrylate, fumed silica, bisphenol-A diglycicyl methacrylate, ethyldimethylaminobenzoate Catalyst: barium aluminoborosilicate glass, ytterbium fluoride, triethylene glycol dimethacrylate, ethoxylated bisphenol-A dimethacrylate, urethane dimethacrylate, fumed silica, bisphenol-A glycidyl methacrylate, hydroxyethylmethacrylate, peppermint oil
Panavia SA Universal	Kuraray Noritake	(Paste A) contains 10-methacryloyloxydecyl dihydrogen phosphate monomer, Bis-GMA, TEGDMA, HEMA, silanated barium glass filler, silanated colloidal silica, dl-Camphorquinone, peroxide, catalysts, and pigments, while the catalyst (Paste B) has hydrophobic aromatic dimethacrylate, silane coupling agent, silanated barium glass filler, aluminum oxide filler, surface-treated sodium fluoride, dl-Camphorquinone, accelerators, and pigments
Clearfil Universal Bond Quick (CU)	Kuraray Noritake	Bis-GMA, HEMA, ethanol, 10-methacryloyloxydecyl dihydrogen phosphate, hydrophilic aliphatic dimethacrylate, colloidal silica, CQ, silane coupling agent, accelerators, initiators, water

**Table 2:** Table Mean shear bond strength and standard deviation of the resin cements to enamel and dentin.

Cement	Shear Bond Strength MPa (SD)		
	Adhesive	Enamel	Dentin
Panavia SA Universal	Yes	17.07 (4.67) A	9.82 (3.04) b
	No	16.79 (4.51) A	6.55 (2.49) c
NX3 Nexus	Yes	17.89 (5.04) A	13.01 (3.28) a
	No	7.05 (3.45) B	0.90 (0.50) d

Groups with a different uppercase letter with enamel or lowercase letter with dentin are significantly different (*P* <0.05)
